# Uncle Sam

**Published:** 1882-05

**Authors:** 


### "UNCLE SAM."

The origin of the cant name "Uncle Sam" was as follows: Immediately after the declaration of war with England, Elbert Anderson, of New York, then a contractor, visited Troy, where was concentrated, and where he purchased, a large quantity of provisions, beef, pork, etc. The inspectors of these articles were Messrs. Ebenezer and Samuel Wilson. The latter gentleman (invariably known as "Uncle Sam") generally superintended in person a large number of workmen, who, on this occasion, were employed in overhauling the provisions purchased by the contractor for the army. The casks were marked "E. A.—U. S." The work fell to the lot of a facetious fellow in the employ of the Messrs. Wilson, who, on being asked by some of his fellow-workmen the meaning of the mark (for the letters U. S., for United States, were then almost entirely new to them), said: "He did not know, unless it meant Elbert Anderson and Uncle Sam"—alluding exclusively, then, to the said "Uncle Sam" Wilson. The joke took among the workmen, and passed currently; and "Uncle Sam" himself being present was occasionally rallied by them on the increasing extent of his possessions. Many of these workmen being of the character denominated "food for powder," were found shortly after following the recruiting drum, and pushing toward the frontier lines for the double purpose of meeting the enemy and of eating the provisions they had lately labored to put in good order. Their old jokes accompanied them, and before the first campaign ended this identical one first appeared in print; it gained favor rapidly, till it penetrated and was recognized in every part of the country, and will no doubt, continue so while the United States remains a nation.

### SANITARY DEPARTMENT OF "HALL'S JOURNAL OF HEALTH."

We are daily receiving requests from our readers to purchase for them "HEALTH FOODS," "FOOD MEDICINES," PURE BAKING POWDERS, and various MEDICINES that our experience has taught us the value of; also standard books; and last but not least, many families rely on us to procure for them PURE BOVINE VACCINE MATTER, which is the only vaccine matter that is always safe. These commissions already keep one person employed much of the time. We have therefore determined to make this a branch of our regular business, and announce ourselves ready to fill orders for GLUTEN OF WHEAT, GLUTEN COFFEE, COOKED FOODS, as CRUSHED WHEAT, BARLEY, OATS, CORN and RYE, also the various preparaions of MALT, and for books, medicines, vaccine matter and any other like articles that can be found in New York market.

E. H. GIBBS & CO.

